# SLATE-PTP: A spine–leaf architecture timestamps and evaluation dataset for IEEE 1588 precision time protocol benchmarking

**DOI:** 10.1016/j.dib.2026.113183

**Published:** 2026-08-20

**Authors:** Alberto Ortega, Víctor Vázquez, Jesús González, Eduardo Ros, Manuel Rodríguez-Álvarez

**Affiliations:** Department of Computer Engineering, Automation, and Robotics, CITIC, University of Granada, 18014, Granada, Spain

**Keywords:** Packet delay variation (pdv), Time telemetry, Network synchronization jitter, IEEE 1588, Hardware latency, Data center fabrics

## Abstract

This data article describes SLATE-PTP, a high-fidelity IEEE 1588 telemetry dataset captured within a physical two-Spine four-Leaf datacenter fabric engineered with PTP-unaware switches. The data collection architecture isolates network-induced impairments from local oscillator drift. While a GNSS-disciplined Grandmaster clock provided a physical PPS reference to stabilize the hardware clock of the capturing network interface card, the LinuxPTP (ptp4l) daemon operated in a non-adjusting, free-running state. Consequently, the recorded four-way timestamps ($t_{1}$, $t_{2}$, $t_{3}$, and $t_{4}$) capture pure network dynamics, including queuing delays, PDV, and structural path asymmetries. To replicate realistic datacenter environments, a hardware traffic generator injected background loads using four distinct packet-size distributions combined with three temporal profiles (constant, stepped, and ramped loads). The dataset comprises structured CSV files containing raw packet telemetry. For reuse potential, the data is coupled with an encapsulated, object-oriented Jupyter Notebook execution pipeline. By leveraging a decoupled interface, this pipeline provides an open-access evaluation sandbox. Researchers can directly ingest these data tracks to train, validate, and benchmark custom statistical packet filters, ML-based delay classifiers, or alternative time offset estimation algorithms without deploying a high-precision physical testbed.

Specifications Table

**Table:** 

Subject	Computer Science
Specific subject area	Empirical PTP network synchronization traces under multi-variable cross-traffic loads inside PTP-unaware Spine–Leaf datacenter networks.
Type of data	Table (Structured CSV files, comprising 45 telemetry tracks with ∼712 thousand total PTP transaction rows and an aggregate uncompressed footprint of ∼50 MB); Code (Jupyter Notebook)
Data collection	Network telemetry was collected in a 2-Spine 4-Leaf topology using MikroTik CRS326 and CRS310 PTP-unaware switches. Synthetic cross-traffic was injected per link using a Cisco TRex generator with Intel E810 NICs. Telemetry was captured on a bare-metal server with an Intel E810 timing NIC, disciplined via external GNSS PPS (FibroLAN Falcon-MX/G). Concurrently, LinuxPTP (ptp4l) ran in a free-running state across two interconnected ports. Raw logs were extracted with Syncarium software.
Data source location	Time-based Technologies and Networks Lab (TIC-117), Department of Computer Engineering, Automation, and Robotics, CITIC, University of Granada, Granada, Spain.
Data accessibility	Repository name: Zenodo Direct URL to data: https://doi.org/10.5281/zenodo.21770837 Data License: Creative Commons Attribution 4.0 International (CC BY 4.0) Code License: MIT License
Related research article	A. Ortega, V. Vázquez, J. González, E. Ros, and M. Rodríguez-Álvarez, “Structured Characterization Methodology for Time Transfer in Packet-Switched Networks: A PTP-Unaware Case Study,” IEEE Transactions on Instrumentation and Measurement, 2026 [1].

## 1 Value of the Data

• **Isolates Pure Network Dynamics**: By running Linux PTP in a free-running state while stabilizing the NIC via a GNSS-disciplined PPS reference, the dataset decouples oscillator drift from network jitter. The resulting ($t_{1}$, $t_{2}$, $t_{3}$, and $t_{4}$) timestamps capture pure queuing delays and path asymmetries.
• **Reflects Real-World Datacenter Traffic**: Data was captured in a physical two-Spine four-Leaf topology using PTP-unaware switches. It features hardware-generated background traffic across four packet distributions (including real-world IMIX and line-rate MTU stress) and three temporal profiles (constant, stepped, ramped), replicating realistic congestion scenarios.
• **Eliminates Expensive Hardware Barriers**: High-precision timing testbeds require costly Grandmasters and network analyzers. This dataset provides an open-access, open-loop sandbox, allowing researchers to train and benchmark statistical filters, ML delay classifiers, or advanced offset estimation mathematical models without deploying physical infrastructure.
• **Enables Multi-Scenario Stress-Testing and Cross-Layer Benchmarking**: By encompassing a rigorous combinatorial matrix of three temporal load behaviors and four disparate packet size distributions, the dataset allows researchers to thoroughly evaluate the boundary conditions and degradation curves of their estimation algorithms under varied congestion severities within an identical structural framework.
• **Ready for Plug-and-Play Reusability**: The data is coupled with an object-oriented Jupyter pipeline. Through a decoupled interface, researchers can inject and evaluate their own message processing and filtering algorithms against the dataset in just a few lines of code.

## 2 Background

High-precision time synchronization via the Precision Time Protocol (PTP) [2] in modern datacenters is fundamentally constrained by Packet Delay Variation (PDV) introduced by PTP-unaware switches in Spine–Leaf topologies. This dataset was compiled to support the multi-stage systematic characterization methodology proposed in the associated research article [1]. The primary methodological motivation behind generating these data was to deterministically isolate network-induced queuing jitter and path asymmetries from local oscillator drift. This isolation was achieved by operating the PTP stack in a free-running state while utilizing a hardware clock disciplined via a Pulse-Per-Second (PPS) reference from a Global Navigation Satellite System (GNSS) [3] receiver as a ground-truth reference bounded within ±20 ns—an uncertainty floor that remains negligible compared to network-level delay variations.

This data article adds value to the published research by open-sourcing the raw, high-fidelity telemetry time series alongside a modular evaluation framework. Crucially, while the original article evaluated specific network workloads, this dataset comprehensively releases those baseline load profiles and expands upon them with two additional traffic models to capture broader congestion scenarios. Replicating complex, hardware-in-the-loop networking testbeds is logistically and financially restrictive. By providing this open-access, reproducible sandbox, this article allows other researchers to benchmark custom packet-selection filters, optimize clock servo controllers, or quantify core asymmetries under identical, extended traffic profiles without deploying physical infrastructure.

## 3 Data Description

**Fig. 1:**
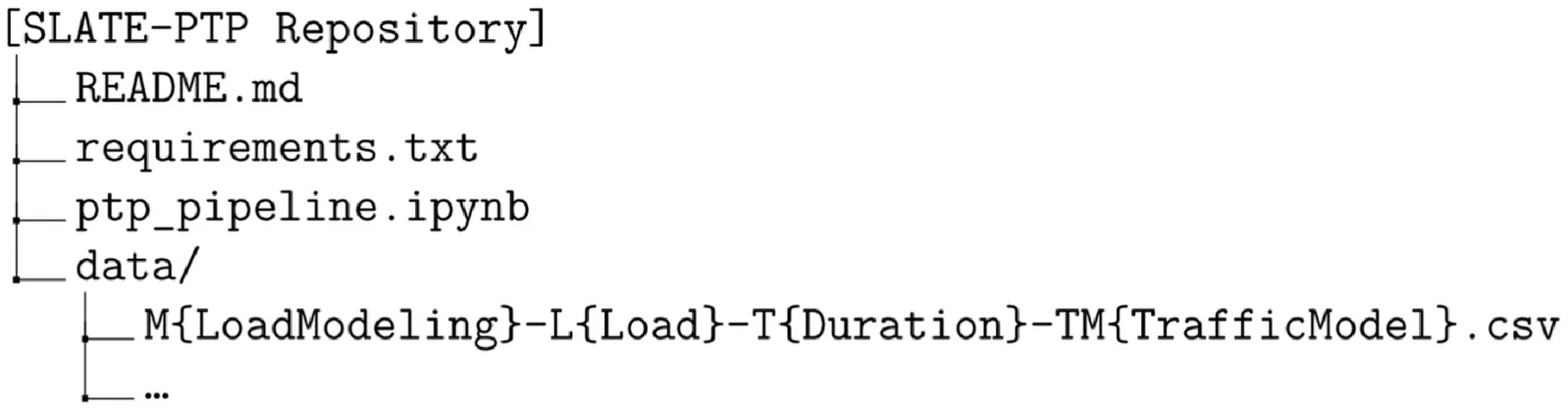
Directory tree structure of the SLATE-PTP dataset repository showing telemetry tracks and pipeline components.

The SLATE-PTP repository is organized into a structured directory tree designed to support automated parsing and reproducibility. The top-level repository contains a data directory (/data), an interactive execution pipeline file (ptp_pipeline.ipynb), and a comprehensive documentation file (README.md). The directory layout is detailed in Fig. 1.

### 3.1 Data directory

In total, the dataset comprises 45 discrete CSV files containing approximately 712 thousand individual PTP timestamp transactions, with a cumulative uncompressed storage footprint of approximately 50 MB across the Zenodo repository.

The /data directory contains raw high-fidelity telemetry tracks stored as individual comma-separated values (.csv) files. Each file represents a discrete experimental run mapping unique background traffic dynamics. Files follow a deterministic naming convention that reflects the experimental configuration parameters:

M{LoadModeling}-L{Load}-T{Duration}-TM{TrafficModel}.csv

where M, L, T, and TM denote the specific operational variables described in Table 1.

**Table 1:** Code definition for dataset file nomenclature.

Parameter	Code Component	Encoded Values / Patterns	Description
M∼	Load Modeling	M1: Constant Load M2: Instantaneous Steps M3: Progressive Ramp	M1: Steady load at specified % for 60 min. M2: 6 discrete load steps (10% ↔ 90%) changing every 5 min. M3: Linear load ramp (10% → 90% → 10%) changing 1% every 5 min over 900 min.
L∼	Traffic Load	Numeric value or range (e.g., L10, L50, L1090)	Represents the background traffic injection volume as a percentage of overall link capacity. Traffic is injected either at a constant rate (e.g., L10 → 10%) or as a dynamic range spanning from 10% to 90% (encoded as L1090 for multi-step/ramp profiles).
T∼	Duration	Numeric integer (e.g., T30, T60, T900)	Explicit total operational time of the experiment in minutes.
TM∼	Traffic Model	TMIMIX: Internet MixTMMTU: Max Trans Unit TMLP: Large Packets TMSP: Small Packets	Frame distribution size proportions (Small [64B] / Medium [576B] / Big [1518B]): • IMIX: 59% / 33% / 8% • MTU: 0% / 0% / 100% • LP: 10% / 15% / 75% • SP: 75% / 15% / 10%

### 3.2 CSV schema and data fields

Each comma-separated telemetry file in the dataset shares a uniform, tabular schema designed to capture low-level network transactions and clock states at nanosecond-level granularity. The rows within these files chronicle sequential PTP message exchanges (specifically Sync and Delay_Req transactions) under the corresponding network stress conditions. The schema structurally decouples network-layer capture metadata from the formal IEEE 1588 four-timestamp mechanism ($t_{1}$, $t_{2}$, $t_{3}$, and $t_{4}$). Table 2 details the precise column specifications, including data type boundaries, physical units, and architectural descriptions for each recorded field.

**Table 2:** Schema definition and data field specifications for .csv telemetry files.

Column Name	Data Type	Units	Description
seq_id	Unsigned Integer	Counter	Incremental sequence identifier mapping consecutive PTP message transactions.
capture_ts	Unsigned Long	Nanoseconds	The Unix epoch timestamp recorded when the NIC captured the frame.
msg_type	String	Categorical	Identifies the PTP frame function; strictly bounded to "sync" or "delay_req".
t1	Unsigned Long	Nanoseconds	Origin timestamp generated by the PTP Server master clock indicating the transmission time of a Sync message.
t2	Unsigned Long	Nanoseconds	Receive timestamp generated by the PTP Client hardware clock indicating the arrival time of a Sync message.
t3	Unsigned Long	Nanoseconds	Send timestamp generated by the PTP Client hardware clock indicating the departure time of a Delay_Req message.
t4	Unsigned Long	Nanoseconds	Receive timestamp generated by the PTP Server master clock indicating the arrival time of a Delay_Req message.

### 3.3 Execution pipeline and documentation

The root repository provides ptp_pipeline.ipynb, a fully self-contained, object-oriented execution infrastructure designed to automate the ingestion, processing, and evaluation of the telemetry dataset. Rather than operating as an active clock synchronization loop, this pipeline acts as an open-loop benchmarking sandbox governed by the master PTPSimulationPipeline class. This component coordinates the parsing of the multi-model data tracks and logs comparative performance metrics based entirely on the raw PTP message timestamps.

A core architectural feature of this framework is its programmatic decoupling of the data ingestion sweep from the specific offset estimation logic. This is achieved through the abstract base interface PTPMessageProcessor, shown in Listing 1, which operates sequentially over the recorded $t_{1}$, $t_{2}$, $t_{3}$, and $t_{4}$ timestamp streams. The pipeline includes two native implementations for baseline comparison: a standard mathematical PTP offset estimator (Ptp4lProcessor) and a sliding-window minimum-delay selection heuristic (LuckyPacketProcessor).

**Table:** 

1 from abc import ABC, abstractmethod
2 from typing import Dict, Any, Optional
3
4 class PTPMessageProcessor(ABC):
5 *"""Abstract Base Class acting as the interface for all PTP mitigation engines."""*
6 @abstractmethod
7 def process(self, msg: Dict[str, Any]) -> Optional[int]:
8 *"""Processes a single PTP* log *frame and returns the calculated clock offset."""*
9 pass

Listing 1: Abstract base interface for PTP mitigation engines.

Crucially, the framework provides an accessible development blueprint to maximize reusability. Researchers can implement custom time-transfer or mitigation algorithms (e.g., Kalman filters, machine learning regressors, or custom heuristics) by following three fundamental steps:

**Table:** 

**1 Inherit from PTPMessageProcessor**: Ensures structural compatibility with the global simulation engine.
**2. Initialize Internal State inside __init__**: Defines tracking registers, sliding windows (deque), or persistent states required across consecutive packet arrivals.
**3. Override the process(self, msg) method**: Parses incoming frames by msg_type ("sync" or "delay_req"), extracts timestamps, executes custom filtering logic, and returns the calculated clock offset in nanoseconds (or None if no update is performed).

Listing 2 illustrates the boilerplate template provided within the repository to enable rapid algorithmic integration.

**Table:** 

1 class YourCustomProcessor(PTPMessageProcessor):
2 *"""Template blueprint for building custom PTP mitigation and filtering engines."""*
3 def __init__(self, custom_param: int = 5):
4 self.custom_param = custom_param
5 self.persistent_state = None
6 self.last_sync_drift = None
7
8 def process(self, msg: Dict[str, Any]) -> Optional[int]:
9 msg_type = msg.get("msg_type")
10
11 *# 1. Handle Forward Path frames (Server -> Client)*
12 if msg_type == "sync":
13 t1, t2 = msg.get("t1"), msg.get("t2")
14 if t1 is not None and t2 is not None:
15 pass *# TODO: Implement forward path logic or data buffering*
16
17 *# 2. Handle Reverse Path frames (Client -> Server)*
18 elif msg_type == "delay_req":
19 t3, t4 = msg.get("t3"), msg.get("t4")
20 if t3 is not None and t4 is not None:
21 pass *# TODO: Implement reverse path logic or window filtering*
22
23 *# 3. Return calculated offset in nanoseconds when ready, otherwise None*
24 return None

Listing 2: Template blueprint for custom algorithm development.

This extensible design allows researchers to inject custom message processing algorithms directly into the automated execution sweep with minimal setup. The pipeline evaluates these estimation streams to output an aggregated results matrix computing Root Mean Square Error (RMSE) metrics against the absolute ground-truth reference, which are then rendered into comparative time-series charts via the plot_mitigation_performance suite.

Complementing this execution engine, the root README.md establishes the technical documentation baseline for the repository, detailing the underlying hardware-in-the-loop testbed topology, Network Interface Card (NIC) and switch specifications. To guarantee long-term execution stability, environment reproducibility, and seamless deployment, the README.md provides step-by-step instructions for setting up an isolated Python virtual environment (venv) using the exact package versions pinned in requirements.txt. These pinned dependencies include Python 3.13, polars v1.43.2, pandas v3.0.5, pyarrow v19.0.1, numpy v2.5.2, matplotlib v3.11.1, seaborn v0.13.2, ipykernel v6.29.5, tqdm v4.70.0, and ipywidgets v8.1.8.

All telemetry data files are released under the Creative Commons Attribution 4.0 International (CC BY 4.0) license, while the Python simulation framework and execution notebooks are distributed under the open-source MIT License.

## 4 Experimental Design, Materials and Methods

The dataset was acquired by following the systematic characterization methodology originally proposed in [1]. While this framework inherently defines a five-stage workflow to evaluate synchronization performance, only the first four phases—*Analytical Infrastructure Context, Traffic Dynamics Modeling, Pre-Experimental Calibration*, and *Experimentation*—apply to this work. Within this execution, the specific parametric criteria and stabilization thresholds designed for the third phase (*Pre-Experimental Calibration*) were replicated exactly as detailed in the original paper to guarantee steady-state baseline conditions. Conversely, the fifth phase, dedicated to the *Final Analysis* and interpretation of results, is explicitly omitted here to comply with the descriptive, non-interpretative nature required for this data paper.

Consequently, the data generation process was structured through these specific procedural steps. First, the network topology, synchronization protocols, and timing hardware dynamics were profiled. Second, realistic application-driven traffic patterns and load intensities were modeled and injected into the fabric. Third, the pre-experimental calibration phase precisely applied the stabilization thresholds from the reference methodology to determine the locked state acquisition time and optimize the observation interval. Finally, during the experimentation phase, controlled data logging was executed under specific load models to capture the log streams containing the raw fundamental hardware timestamps.

### 4.1 Analytical infrastructure context

#### 4.1.1 Network description

**Fig. 2:**
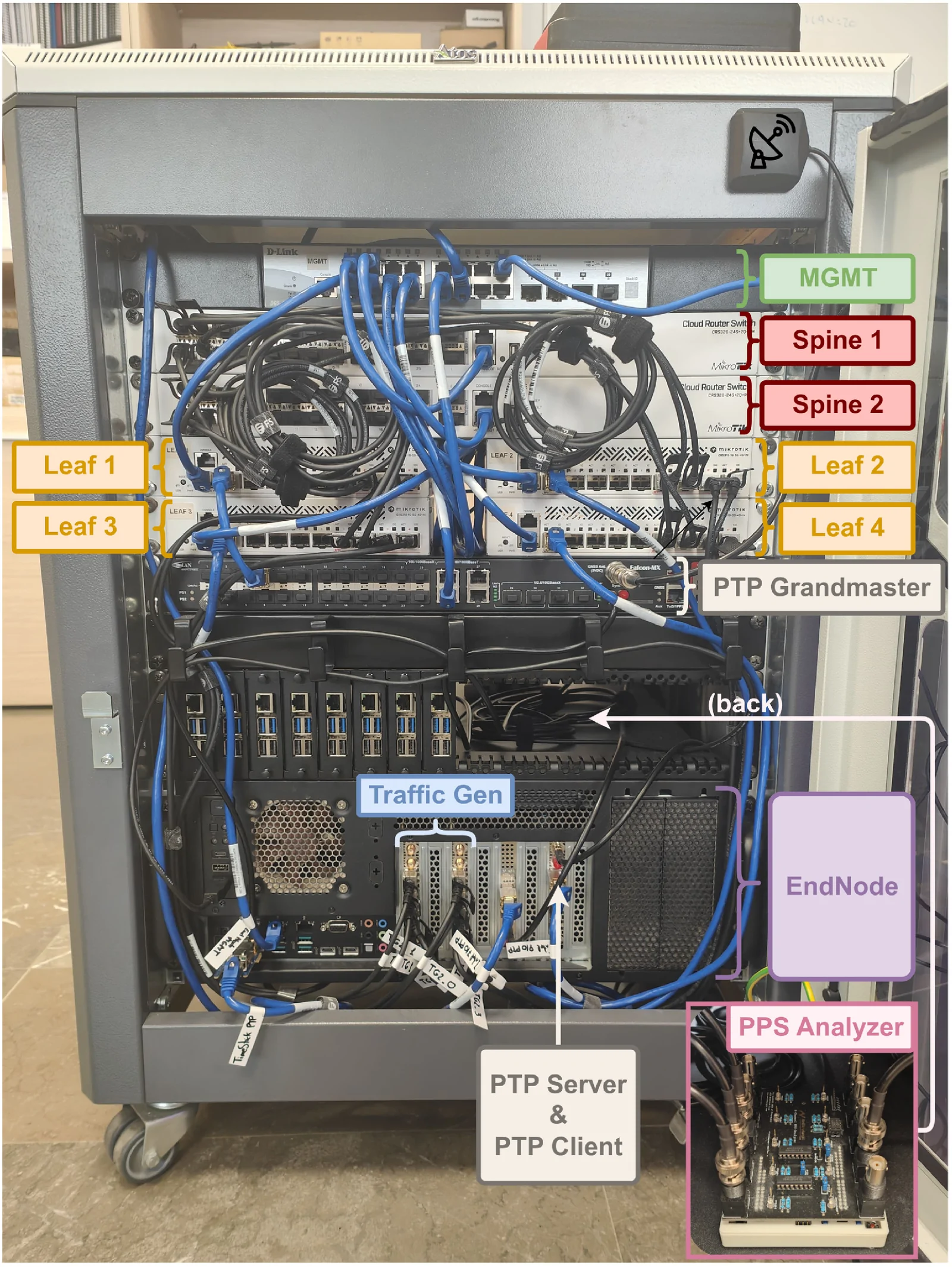
Laboratory rack testbed.

**Fig. 3:**
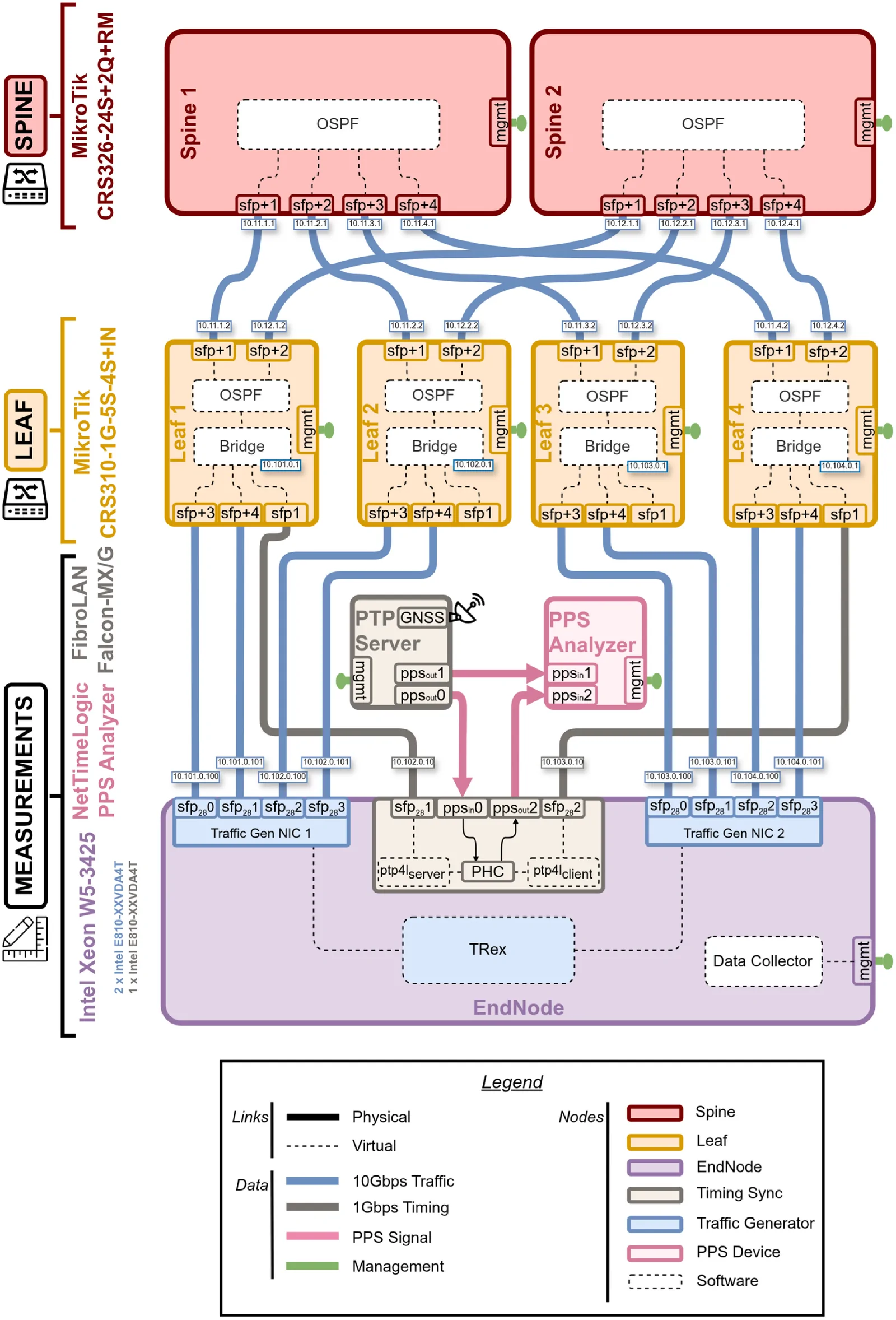
Spine–Leaf testbed complete design.

The experimental data was acquired using a laboratory rack testbed configured in a two-Spine, four-Leaf network architecture Fig. 2, Fig. 3. This structure isolates communication layers, where Leaf switches act as access nodes for end devices and Spine switches aggregate traffic between Leaf switches. The testbed operates on a Layer 3 OSI model using the Open Shortest Path First (OSPF) routing protocol [4] coupled with Equal-Cost Multipath (ECMP) [5] for multipath forwarding. Background network load was injected using the TRex Traffic Generator [6] at the end node, distributing concurrent traffic flows across all equal-cost paths. All physical hardware components and infrastructure layout details are structured in Table 3 and mapped in Fig. 3.

**Table 3:** Network Hardware Features.

Role	Hardware	Features
Spine Switches	MikroTik CRS326–24S+2Q+RM [7]	24 SFP+, 2 QSFP+, Marvell 98DX8332
Leaf Switches	MikroTik CRS310–1G-5S-4S+IN [8]	4 SFP+, 5 SFP, Marvell 98DX226S
End Node & Traffic Gen	Intel Xeon W5–3425 [9] & Intel E810-XXVDA4T [10]	Ubuntu 24.04 & TRex Traffic Generator v3.06 [6]
Links	DAC 10 Gbps Data & Cat6a 1 Gbps Timing	SFP 1 Gbps Downlinks

#### 4.1.2 Synchronization protocol contextualization

To observe raw synchronization dynamics without software servo interference, the physical PTP Hardware Clock (PHC) of the timing NIC is disciplined solely via an external GNSS-derived PPS reference signal. This physical discipline maintains baseline clock stability within a verified ±20 ns ground-truth error floor, continuously monitored by a NetTimeLogic PPS Analyzer [11]. Topologically, the timing NIC operates concurrently as a ptp4l server on one physical port and a strictly *free-running* ptp4l client on another, interconnected through the Spine–Leaf network fabric.

This *free-running* client ptp4l daemon functions as a passive observer that never steers or adjusts the underlying PHC, as it is physically anchored by the external PPS signal. Therefore, since the PTP transaction occurs between two ports sharing the exact same PHC, the true physical offset is strictly zero (0 ns), and any non-zero offset estimation processed by the ptp4l client directly quantifies the estimation error introduced by network-induced PDV and queuing asymmetries.

To collect the PTP estimations, the dataset captures the four fundamental raw hardware timestamps—$t_{1}$, and $t_{2}$ from Sync/Follow_Up sequences, and $t_{3}$ and $t_{4}$ from Delay_Req/Delay_Resp exchanges. These raw timestamps and standard ptp4l log streams are collected following the software configuration profile detailed in Table 4.

**Table 4:** Main configuration parameters used for ptp4l.

Parameter	Value	Description
ptp4l version	v3.1.1	Client compatibility
Servo function	PI	Proportional-Integral
$K_{p}$	0.7	Proportional gain
$K_{i}$	0.3	Integral gain
Delay mechanism	E2E	End-to-End delay
Transport	UDP/IPv4	PTP transport
Domain number	0	Domain in use
Priority 1 & 2	255	Lowest priority (Client/Follower)
logAnnounceInterval	0	Every 1 s
logSyncInterval	0	Every 1 s
logMinDelayReqInterval	0	Every 1 s
Time Stamping	Hardware	Hardware timestamping enabled
free-running	1	Not adjusting clock
Sync mode	Two-step	twoStepFlag=1
Intel E810-XXVDA4T Driver	v1.17	Linux 6.8.0 in-tree

**Technical Note on Message Scheduling**: The recorded PTP exchange does not follow a strict 1:1 alternating pattern, as it contains only the asynchronous message events and their respective timestamps. While Sync packets maintain a deterministic 1-second transmission interval, Delay_Req messages are inherently non-uniform. In compliance with the IEEE 1588 End-to-End mechanism, ptp4l randomizes Delay_Req transmissions (varying between 0 and 2 s for a logMinDelayReqInterval of 0) to avoid network queuing effects. Consequently, the dataset structure consists of independent, asynchronous timestamp entries where multiple consecutive Sync events (providing $t_{1}$, $t_{2}$) may appear between successive Delay_Req events (providing $t_{3}$, $t_{4}$), or vice versa.

### 4.2 Timing hardware specification

The core specifications of the timing infrastructure are detailed in Table 5. The PTP Grandmaster consists of a FibroLAN Falcon-MX/G unit [12] equipped with an Oven Controlled Crystal Oscillator (OCXO) [13] disciplined by a GNSS reference [3], maintaining a frequency stability of $10^{-12}$ and a phase accuracy within ±100 ns relative to GNSS.

**Table 5:** Timing hardware features.

Role	Hardware	Clock Features
PTP Grandmaster	FibroLAN Falcon-MX/G [12]	OCXO & GNSS
Timing NIC (PTP Server and Client)	Intel E810-XXVDA4T [10]	high-precision oscillator & DPLL

The experimental evaluation is conducted on a bare-metal node running Ubuntu 24.04 and the ptp4l Linux PTP implementation [14]. The PTP server and client are consolidated into a single high-performance NIC, specifically the Intel Ethernet E810-XXVDA4T [10]. This card is equipped with an internal PHC that handles hardware-level timestamping [15], driven by an onboard high-precision oscillator and managed via a Digital Phase-Locked Loop (DPLL) control system [16]. PTP server and client workloads are segmented using two independent ports on this NIC. Intermediate network switches are PTP-unaware, limiting timestamp generation exclusively to the end-hosts.

The data collection infrastructure relies on a closed-loop measurement framework managed by Syncarium [17], an automated data collection tool developed to simplify the configuration, deployment, and monitoring of time synchronization platforms in distributed environments.

### 4.3 Traffic dynamics modeling

Ethernet traffic is injected into the topology using a traffic generator.

### 4.4 Application-driven traffic patterns

The traffic models selected represent the types of traffic generated by applications typically deployed in data center topologies. The models shown in Table 1 capture representative behaviors:

• **Internet Mix (IMIX)**: Represents a heterogeneous traffic profile matching standard internet packet distributions. This model is critical for evaluating data center front-end tiers and edge switches facing public user-generated requests, balancing short control messages with larger payload transfers.
• **Maximum Transmission Unit (MTU)**: Composed exclusively of maximum-sized Ethernet frames (1518 bytes). This profile simulates bulk data transfer applications operating at maximum link efficiency, such as Storage Area Networks (SAN), intra-datacenter replication, and large-scale backup procedures.
• **Small Packet (SP)**: Characterized by a predominance of small packets, representing applications with frequent short exchanges between nodes, such as distributed databases, key-value stores, or heavy microservices.
• **Large Packet (LP)**: Dominated by large packets, representing applications involving heavy data transfers, such as distributed video-streaming services, big data analytics, or machine learning training workloads.

### 4.5 Traffic intensity levels

Traffic injection rates were systematically scaled as exact percentages of the Leaf→Spine uplink capacity. Given that these physical interfaces operate at a nominal speed of 10 Gbps, a targeted load level (e.g., 25%) translates to a constant throughput of 2.5 Gbps per direction. The TRex traffic generation platform was configured to enforce strictly bidirectional, full-duplex continuous flows across all topology links; each uplink simultaneously transmitted and received the designated bandwidth percentage. This continuous injection model prevents transient traffic bursts, guarantees deterministic background stress, and ensures stable, highly reproducible load conditions throughout each experimental run.

### 4.6 Experimentation

The experimental scenarios designed for this dataset are based on the core evaluation frameworks originally proposed in [1]. These scenarios adapt the standardized testing philosophy of Appendix VI in the ITU-T G.8261/Y.1361 recommendation [18] to datacenter environments by leveraging the specific load modeling and traffic dynamics detailed in the previous sections. Specifically, the experimental phase is executed across three distinct test cases, fully aligned with the load models defined in Table 1:

• **Constant Load Experiment (M1**): This experiment evaluates the steady-state behavior and long-term stability of client synchronization under unvaried network conditions. Background traffic is injected at fixed operational levels to determine how different traffic profiles affect performance over extended periods. Steady load at specified % for 60 min.
• **Instantaneous Steps Experiment (M2)**: This scenario examines the resilience, transient response, and recovery path of the timing system when subjected to sudden traffic variations. The network fabric undergoes sharp, discrete load transitions, capturing the behavior during abrupt saturation and decompression cycles. 6 discrete load steps (10% ↔ 90%) changing every 5 min.
• **Progressive Ramp Experiment (M3)**: This experiment assesses the synchronization system’s tracking capability and sensitivity under slowly and linearly varying traffic conditions. Background traffic scales continuously, allowing the observation of accumulation effects. Linear load ramp (10% → 90% → 10%) changing 1% every 5 min over 900 min.

## Limitations

The dataset presents specific limitations regarding its infrastructural scope and environmental context during data collection:

• **Thermal and Environmental Control:** Tests were conducted in a standard laboratory without active climate control. While long-duration experiments could suggest potential thermal drift, this effect is neutralized at the hardware layer. Although LinuxPTP (ptp4l) ran in software *free-running* mode to avoid servo artifacts, the NIC hardware clock was continuously disciplined by a GNSS-derived PPS reference. This hardware-level discipline absorbs local thermal drift and bounds timing error within a tight ±20 ns floor, ensuring telemetry ($t_{1},t_{2},t_{3},t_{4}$) strictly reflects pure network-induced delays.

## Ethics Statement

The authors have read and follow the ethical requirements for publication in Data in Brief and confirm that the current work does not involve human subjects, animal experiments, or any data collected from social media platforms.

## CRediT authorship contribution statement

**Alberto Ortega:** Conceptualization, Methodology, Software, Validation, Formal analysis, Investigation, Data curation, Writing – original draft, Writing – review & editing, Visualization. **Víctor Vázquez:** Conceptualization, Methodology, Software, Validation, Formal analysis, Data curation, Writing – review & editing. **Jesús González:** Writing – review & editing, Supervision. **Eduardo Ros:** Writing – review & editing, Supervision, Resources, Project administration. **Manuel Rodríguez-Álvarez:** Writing – review & editing, Supervision, Resources, Project administration, Funding acquisition.
